# Overinterpretation of findings in machine learning prediction model studies in oncology: a systematic review

**DOI:** 10.1016/j.jclinepi.2023.03.012

**Published:** 2023-05

**Authors:** Paula Dhiman, Jie Ma, Constanza L. Andaur Navarro, Benjamin Speich, Garrett Bullock, Johanna A.A. Damen, Lotty Hooft, Shona Kirtley, Richard D. Riley, Ben Van Calster, Karel G.M. Moons, Gary S. Collins

**Affiliations:** a Centre for Statistics in Medicine, Nuffield Department of Orthopaedics, Rheumatology and Musculoskeletal Sciences, University of Oxford, Oxford OX3 7LD, UK; b NIHR Oxford Biomedical Research Centre, Oxford University Hospitals NHS Foundation Trust, Oxford, UK; c Julius Center for Health Sciences and Primary Care, University Medical Center Utrecht, Utrecht University, Utrecht, The Netherlands; d Meta-Research Centre, Department of Clinical Research, University Hospital Basel, University of Basel, Basel, Switzerland; e Nuffield Department of Orthopaedics, Rheumatology, and Musculoskeletal Sciences, University of Oxford, Oxford, UK; f Centre for Prognosis Research, School of Medicine, Keele University, Staffordshire, UK, ST5 5BG; g Department of Development and Regeneration, KU Leuven, Leuven, Belgium; h Department of Biomedical Data Sciences, Leiden University Medical Center, Leiden, the Netherlands; i EPI-centre, KU Leuven, Leuven, Belgium

**Keywords:** Prediction model, Machine learning, Spin, Oncology, Prognosis, Statistical learning, Artificial intelligence

## Abstract

**Objectives:**

In biomedical research, spin is the overinterpretation of findings, and it is a growing concern. To date, the presence of spin has not been evaluated in prognostic model research in oncology, including studies developing and validating models for individualized risk prediction.

**Study Design and Setting:**

We conducted a systematic review, searching MEDLINE and EMBASE for oncology-related studies that developed and validated a prognostic model using machine learning published between 1st January, 2019, and 5th September, 2019. We used existing spin frameworks and described areas of highly suggestive spin practices.

**Results:**

We included 62 publications (including 152 developed models; 37 validated models). Reporting was inconsistent between methods and the results in 27% of studies due to additional analysis and selective reporting. Thirty-two studies (out of 36 applicable studies) reported comparisons between developed models in their discussion and predominantly used discrimination measures to support their claims (78%). Thirty-five studies (56%) used an overly strong or leading word in their title, abstract, results, discussion, or conclusion.

**Conclusion:**

The potential for spin needs to be considered when reading, interpreting, and using studies that developed and validated prognostic models in oncology. Researchers should carefully report their prognostic model research using words that reflect their actual results and strength of evidence.

**What's new?**

**Key findings**
• We found areas highly suggestive of spin in prediction model research.

**What this adds to what was known?**
• Specific areas highly suggestive of spin included inconsistent reporting between the methods and results sections due to selective reporting and additional analysis and use of overly strong and leading words in the title, abstract, results, discussion, or conclusion sections, especially when making (often unfair) comparisons between nonregression (e.g., neural networks) and regression machine learning methods (e.g., logistic regression).

**What is the implication and what should change now?**
• The potential for spin needs to be considered when writing, reading, and interpreting studies that developed and validated prediction models in oncology.
• Researchers should ensure any comparison between developed models and between developed and existing published models are fair by fully reporting methods.
• Researchers should carefully report their prediction model research using words that reflect their actual results and strength of evidence.

## 1 Introduction

Prediction models are commonly used in health care with a view to support medical decision making, informing individual diagnoses, prognoses, and risk prediction [1,2]. They are especially used in oncology where they help inform cancer diagnoses, prognoses once diagnosed with cancer, and risk of developing cancer in the future [[3], [4], [5]]. Prediction models can increase the speed of diagnosis of a cancer, help guide treatment plans for a patient and guide risk reduction strategies [6].

Given the use and popularity of prediction models, there is a constant drive to improve their predictive performance. One area needing improvement is the methods used by researchers to analyze data and develop the prediction models. Machine learning has rapidly risen in popularity in all areas of research but especially prediction model research, promising improved and personalized prediction. However, this promise is largely, barring a handful of exceptions, yet to be delivered and questions have arisen regarding the hype of machine learning [7].

A growing concern in biomedical research is that of ‘spin,’ which relates to the overinterpretation of study findings. Spin, which can be both intentional and unintentional, is defined as ‘specific reporting that fails to faithfully reflect the nature and range of findings and that could affect the impression that the results produce in readers, a way to distort science reporting without actually lying’ [8]. Spin can impact researchers and clinicians, bias readers' judgments, and negatively influence future research and clinical practice. Findings may not translate to clinical practice as well as suggested, potentially harming patients if a model is implemented based on claims that are too optimistic. The reach of spin also extends to the general public, who might be exposed and more vulnerable to embellished press releases from primary studies with spin [9], resulting in potentially more direct but unnecessary behavior change.

Studies evaluating spin in medical research have largely focused on randomized and nonrandomized controlled trials [10,11], systematic reviews [12], prognostic factors, and diagnostic test accuracy studies [[12], [13], [14], [15]]. To date, no studies have evaluated the prevalence of spin and how it is manifested in prediction model research in oncology, though numerous studies highlighting poor reporting quality, high risk of bias, and poor methodological conduct [[16], [17], [18], [19]], suggests there is potentially high risk of spin in these studies, given the ongoing machine learning hype.

In this study, we reviewed and described areas highly suggestive of spin in prediction model research studies that use author-defined machine learning methods in low dimensional settings in oncology. We specifically reviewed the spin practice in machine learning studies given the level of hype that has been established in this area and focus the review on oncology given the large number and breadth of prediction models and modeling approached being developed in this area and to specifically include prediction models using diverse modeling approaches and for binary and time-to-event outcomes. Our findings will inform the development of the transparent reporting of a multivariable prediction model for individual prognosis or diagnosis (TRIPOD)-artificial intelligence (AI) reporting guideline [20].

## 2 Methods

### 2.1 Protocol registration and reporting standards

This study was registered under an umbrella review with PROSPERO (ID: CRD42019140361) [21] that consists of four distinct studies to evaluate [1] quality of reporting [2,22], risk of bias [3], methodological conduct [23], and [4] spin or overinterpretation (present paper). In the present paper, we build on findings in the quality of reporting and methodological conduct papers and provide further detail about reporting (including use of an appropriate reporting guideline and selective reporting) and its implications specifically on spin. We reported our study following the preferred reporting items for systematic reviews and meta-analyses (PRISMA) guideline and its extension for reporting literature searches in systematic reviews (PRISMA-S) [[24], [25], [26]].

### 2.2 Information sources

We searched the MEDLINE (via OVID) and Embase (via OVID) medical literature databases for prognostic model studies developed using machine learning methods within the oncology field and published between 1st January, 2019, to 5 September, 2019 (the date that the search was conducted).

The full search strategies for both databases are provided in Supplementary Tables 1 and 2. The search terms included relevant Mesh and EMTREE headings and free-text terms. We searched in the title, abstract, or keyword fields, for general modeling terms (such as “machine learning” and “deep learning”), more specific machine learning modeling terms (such as “random forest,” “support vector machine,” and “neural networks”), cancer terms (such as “cancer”, “malignant,” and “carcinoma”), prediction-related search terms (such as “prediction”, “prognostic,” and “risk of”), and specific model performance terms (such as “discrimination” and “calibration”). Modeling, cancer, and prediction terms were combined with ‘and’ to retrieve publications meeting all three sets of search criteria. The search was limited to retrieve studies published in 2019 only to ensure that a contemporary sample of studies was assessed in the review. Apart from the date range specified, no other limits were applied to the search. An information specialist (SK) was involved in the development of the search strategy for both databases.

### 2.3 Eligibility criteria

We included published studies developing a prediction model for individualized prediction using machine learning methods, as defined by authors of the primary report, within the oncology field in 2019. We included studies developing a prognostic model if the modeling method was defined as machine learning by the authors of the primary report. For example, studies using logistic regression were included if they were explicitly described as machine learning by the primary study authors anywhere in the primary report, else it were excluded. We took this approach because the boundary between machine learning and statistical (regression-based) methods for prediction is unclear and often cultural rather than based on specific methods [27]. While some methods, such as neural networks, typically fall into machine learning taxonomy, other methods, such as logistic regression, are frequently ascribed to both domains.

We included studies developing a model using at least two or more predictors (prognostic factors) to predict a health outcome (with no restrictions on the outcome format). No restriction was placed on study design. We excluded studies that only evaluated the performance of an existing prediction model (e.g., an external validation study without any model development). To retrieve a sample of studies that reflect low- dimensional settings, we excluded imaging studies, or studies using imaging parameters as candidate predictors in the model; speech recognition/voice pattern studies, or studies using speech parameters as candidate predictors; genetic studies, or studies using genetic risk factors as candidate predictors; and molecular studies, or studies using molecular markers as candidate predictors. We also excluded risk or prognostic factor studies, secondary research (e.g., reviews of prediction models), and conference abstracts. Studies were limited to English language studies only.

### 2.4 Study selection, data extraction, and data management

Publications identified from MEDLINE and Embase were imported into Endnote reference software, where they were deduplicated, and then imported into the Rayyan web application, where they were screened [28,29].

Two independent researchers (PD, JM) screened the titles and abstracts of the identified publications. Two independent researchers, from a combination of five reviewers (PD, JM, GB, BS, and CLAN) reviewed the full text for potentially eligible publications and performed a double data extraction of eligible publications. One researcher screened all publications (PD) and four researchers collectively screened the same publications (JM, GB, BS, and CLAN). Disagreements were discussed and adjudicated by a third reviewer (GSC), where necessary.

A formal framework has yet to be developed to detect and classify spin in prediction model studies. We therefore described areas highly suggestive of spin and assessed the presence of possible spin in the included studies, predominantly guided by a classification scheme developed and used by Kempf et al. to assess spin in prognostic factor studies in oncology [30], guidance developed by Boutron et al. [8], and the TRIPOD reporting guideline [31,32]. The classification of spin by Kempf et al. and guidance from Boutron et al. highlight three domains for spin: misleading reporting (e.g., selective, incomplete, and mis-reporting of methods and results), misleading interpretation (e.g., unreliable statistical analysis and inappropriate inference) and misleading extrapolation of the results (claiming irrelevant clinical applicability, ignoring uncertainty). We developed the data extraction form to include items which would extract information that would evaluate studies in these three domains.

We reviewed individual sections of the published paper for the following information:

1. whether the published studies did not adhere to a reporting guideline or used an inappropriate guideline (to assess possible misleading reporting)
2. did not make a protocol available–published or unpublished (to assess possible misleading reporting)
3. the inappropriate use of strong and leading words in the title, abstract, results, discussion, and conclusion sections to describe model or model performance–we searched for words including ‘novel,’ ‘excellent,’ ‘accurate,’ ‘optimal,’ ‘perfect.’ and ‘significant.’ We also included an ‘other’ option to capture additional strong and leading words that may indicate spin. If a strong and leading word was found, we reviewed the context of the complete statement (including study aims, sample size calculations, methodology, results, and strength of evidence) to support the suggestion of spin and reported supporting example statements of spin (to assess possible misleading interpretation and extrapolation of the results)
4. whether axes of included figures had been squashed or truncated (to assess possible misleading reporting, misleading interpretation)
5. whether inappropriate and unjustified comparisons were made between the models developed in the present study or with previously published models in the discussion section–we described if developed models were found to be better or worse than previously published models and if study authors provided reasoning for the difference in model performance (to assess possible misleading reporting, misleading interpretation, and misleading extrapolation)
6. inconsistency in reporting between the methods and results sections and the results and conclusion sections in the main text (to assess possible misleading reporting, misleading interpretation, and misleading extrapolation)
7. inconsistency in reporting in the abstract and main text (to assess possible misleading reporting, misleading interpretation, and misleading extrapolation)

The data extraction form was implemented using Research Data Capture (REDCap) software [33].

### 2.5 Data items and summary measures and synthesis of results

Descriptive information was extracted about each publication, including cancer type, study type, data source/study design, target population, type of prediction outcome, number and type of machine learning models used, setting, intended use and aim of the prognostic model. Items were extracted separately for the development and, if done, for validation of the models. Extracted data items to capture possible spin in the included studies can be found in Supplementary Table 3.

Findings were summarized using descriptive statistics and a narrative synthesis. Analysis and synthesis of data were presented overall. All analyses were carried out in Stata v15 [34].

## 3 Results

**Fig. 1:**
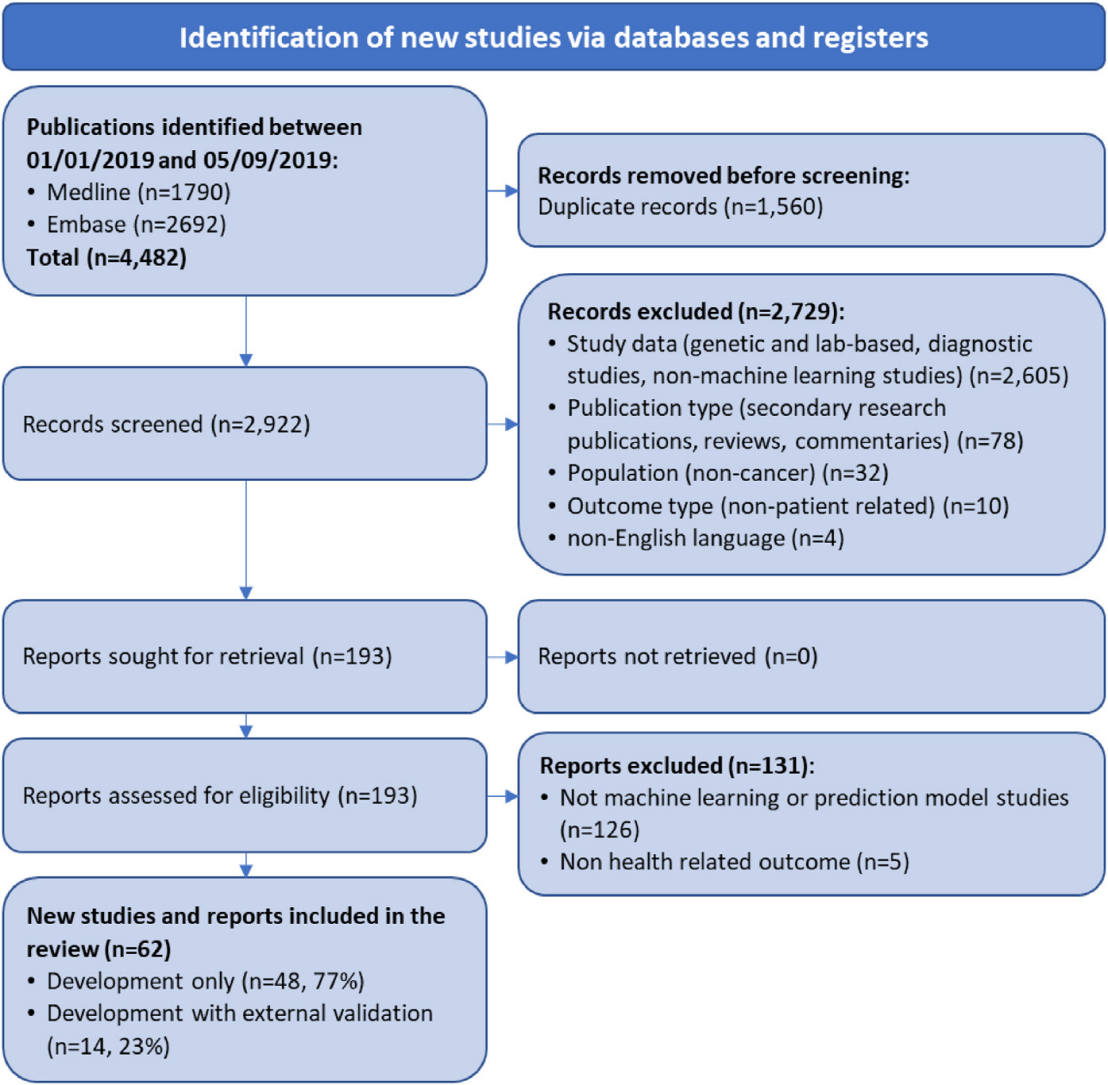
Flow diagram of included studies.

The search in MEDLINE and Embase retrieved 2,922 unique studies published between 1 January 2019 and 5 September 2019. Title and abstract screening excluded 2,729 publications and full text screening excluded a further 131 publication that did not meet the eligibility criteria. 62 publications were included in our review, of which 77% (*n* = 48/62) were development only studies and 23% (*n* = 14/62) were development and external validation studies (Figure 1). Citations for all included studies are provided in Supplementary Table 4.

### 3.1 Study characteristics

Prognostic models were predominantly developed or externally validated for lung cancer (*n* = 8/62, 13%), breast cancer (*n* = 6/62, 10%), and colorectal cancer (*n* = 6/62, 10%) (Table 1). The target population was most often cancer patients (*n* = 55/62, 89%); six studies (10%) predicted cancer outcomes in the general population and for one study (1%) where the target population was unclear.

**Table 1:** Study characteristics for all included studies

Study characteristics	All studies (*n* = 62) *n* (%)
Cancer type	
Lung	8 (13)
Breast	6 (10)
Colon/colorectal/rectal	6 (10)
Gynecological (inc. cervical, ovarian, and endometrial)	6 (10)
Head and neck	5 (8)
Prostate/penile	5 (8)
Brain (inc. meningioma and glioblastoma)	5 (8)
Spinal	4 (6)
Pancreatic	3 (5)
Gastric	3 (5)
Oral (inc. nasopharyngeal carcinoma)	3 (5)
Liver	2 (3)
Skin (inc. melanoma)	2 (3)
Other^a^	4 (6)
Target population	
Cancer patients	55 (89)
General population	6 (10)
Unclear	1 (1)
Outcome	
Binary	48 (77)
Time to event	11 (18)
Multinomial	2 (3)
Continuous	1 (2)
Data source	
Registry	21 (34)
Retrospective cohort	14 (23)
Prospective cohort	9 (15)
Routine care database	9 (15)
Unclear	5 (8)
Other^b^	3 (5)
Randomized controlled trial	1 (2)
Setting	
Secondary care	36 (58)
Tertiary care	10 (16)
Unclear	6 (10)
General population	5 (8)
Other^c^	3 (5)
Primary care	2 (3)
Geographic location	
USA	21 (34)
Europe	13 (21)
Unclear	12 (19)
Asia	8 (13)
Canada	3 (5)
South America	2 (3)
Europe, North America, and Australia	1 (2)
Europe, South America	1 (2)
South Asia, USA	1 (2)
Intended user	
Health-care providers	34 (55)
Unclear	19 (31)
Health care providers and patient/public	4 (6)
Public/patients	2 (3)
Health-care providers and researchers	2 (3)
Researchers	1 (2)
Aim of model	
Predict a risk	36 (58)
Classify patients	25 (40)
Predict length of stay (continuous outcome)	1 (2)

a Other includes peritoneal carcinomatosis, incurable cancer (various), leukemia, and malignant peripheral nerve sheath tumor.

b Other includes audit, survey, and a combination data source of hospital and research data and a registry.

c Other includes combination of hospitals, hospices and nursing homes, NTT medical center in Tokyo, and combination of primary and tertiary care.

Models were often developed using registry data (*n* = 21/62, 34%) and on data from the United States (*n* = 21/62, 34%). Models were predominantly intended to be used by health-care providers (*n* = 34/62, 55%). Fifty-six articles (*n* = 56/62, 90%) included a conflicts of interest section, and most studies declared no conflicts of interest (*n* = 45/56, 80%).

### 3.2 Reporting consistency between methods, results and conclusions in the main text

Results about reporting consistency between methods, results, and conclusions in the main text are shown in Table 2. A published or unpublished protocol was not referenced or available for any study. Five studies stated using a reporting guideline (*n* = 5/62, 8%) [[35], [36], [37], [38], [39]]. Two studies used STROBE, designed for reporting cohort studies (including one study that also reported using a reporting guideline for machine learning studies by Luo et al. [40]) [36,37]. Three studies used TRIPOD (including two studies that also stated using a reporting guideline for machine learning studies by Luo et al.) [35,38,39].

**Table 2:** Use of a reporting guideline and reporting consistency between the methods and results sections

Item	*N*	%
Is adherence to a reporting guideline mentioned?		
Yes	5	8.1
No	57	91.9
Are results consistent with what was specified in the methods?		
Yes	47	75.8
No	15	24.2
Is there any subgroup analysis specified?		
Yes	6	9.7
Is reporting of results of subgroup analysis consistent with what was prespecified?		
Yes	*3*	
No	*3*	
Not applicable	56	90.3
Is there any sensitivity analysis specified?		
Yes	7	11.3
Is reporting of results of sensitivity analysis consistent with what was prespecified?		
Yes	*2*	
No	*5*	
Not applicable	55	88.7
Consistent use of categorization of continuous predictors?		
Yes	14	22.6
No	4	6.5
Unclear	37	59.7
Not applicable^a^	7	11.3

Italic values are a subset to the preceeding question and value.

a includes studies that did not have continuous predictors or did not categorise them.

Results were found to be inconsistent with analyses that were specified in the methods sections of the respective articles in almost a quarter of studies (*n* = 15/62, 24%). Thirteen of these studies (*n* = 13/15, 87%) conducted additional analyses (including comparing machine learning algorithms to regression-based models) that were not specified in the methods section of the study [35,37,38,[41], [42], [43], [44], [45], [46], [47], [48], [49], [50]]. For example, one study did not specify the comparison between a XGBoost machine learning algorithm and a logistic regression model in their methods section [50]. A further two studies selectively their results [51,52]; one study only reported two out of the five machine learning algorithms they had planned to develop [51]; one study only reported their ‘most robust’ model out of the four models they had specified to develop in their methods [52].

Six studies (*n* = 6/62, 10%) reported at least one subgroup analysis [35,46,49,[53], [54], [55]], however, three studies did not report their results consistent with what was specified in the methods section [35,49,54]. Similarly, sensitivity analyses were reported in seven studies (*n* = 7/62, 11%) [37,47,49,[56], [57], [58], [59]], of which five studies did not report their results consistently to what was prespecified [37,47,49,57,58].

Fifty-five studies categorized some or all of their continuous predictors (*n* = 55/62, 89%), however, categorization methods were undefined, model-dependent but not clearly defined, or unreported for over two-thirds of studies (*n* = 37/55, 67%). Four studies did not use consistent categorization of continuous predictors; in one study, categorization was not consistently used throughout the analysis [60] and in three studies, cut-points to define categorization changed throughout the analysis when developing decision trees [37,41,54].

### 3.3 Model comparisons in the discussion

Thirty-two studies (out of 36 applicable studies that developed more than one model, or 89%) made comparisons between the models they developed in the discussion section. None of these studies reported model comparison as an aim of their study, and none reported a sample size calculation to power their study to detect differences in performance. Twenty of these studies (*n* = 20/32, 63%) identified the better-performing model in the discussion but did not base this on an explicit comparison to the other developed models and were reported in isolation from other model results. For example, a study reported in their discussion that ‘the SGB [stochastic gradient boosting] model achieved superior performance on both cross-validation of the training set and testing in the independent holdout set,’ without reporting the comparison models [39]. However, twelve studies (*n* = 12/32, 38%) reported that their ML model was the better-performing model with a direct comparison to a regression model. For example, a study reported that ‘ANN [artificial neural network] models showed better discriminatory performance than multivariable logistic regression models’ [37].

The area under the curve (AUC) was most commonly used to support these claims (*n* = 25/32, 78%), and classification measures (e.g., sensitivity specificity, accuracy) were used in five studies (*n* = 5/32, 16%). Calibration was reported in addition to discrimination in four studies to support model comparisons (*n* = 4/32, 13%) [38,39,55,61].

Thirty-two studies (*n* = 32/62, 52%) also made a reference to and compared their developed models to previously published models (24 development-only studies and eight development with validation studies). Only one development and validation study formally compared their developed models to a published model in a validation analysis; all other studies compared the model performance values of developed models to model performance values that were reported with the previously published model in its origin paper. Nineteen studies (*n* = 19/32, 59%) declared their developed models to be better than previously published models (13 development-only studies and six development with validation studies); four studies did not report if their model was better or worse (3 development-only studies and 1 development with validation study), and for nine studies, it was unclear (8 development-only studies and 1 development with validation study). Of the 19 studies declaring their models better than previously published models, only half gave reasons for the difference in performance, limiting the evaluation of their applicability (*n* = 9/19, 47%).

### 3.4 Reporting in the abstract

Inconsistent reporting was found in the abstract of seven studies (*n* = 7/62, 11%) that included additional results not found in the main text [62], used different age categories to what was used in the main text [41], selectively reported the models that were developed [52,63], and misreported the conclusion [47], the sample size (likely reporting the sample size before eligibility criteria was applied) [64] and study setting [50] compared to the main text.

Forty-six study abstracts (*n* = 46/62, 74%) reported the number of models to be developed, which ranged from 1 to 29 models (median: 1 model, IQR: 1 to 3), compared to a range of 1 to 6 models that were reported and developed in the main text of the studies (median: 2 models, IQR: 1 to 4) (Table 3).

**Table 3:** Reporting consistency between the main text and abstract

Item	Abstract		Main text	
Is there a reference or mention to compare the machine learning technique with traditional statistical methods in the developed models? *n* = 36	*N*	%	*N*	%
Yes	13	36.1	32	88.9
No	23	63.9	4	11.1
Not applicable (one 1 model developed), *n* = 62	26 (41.9)		26 (49.1)	
Which model performance measures were reported?^a^				
Discrimination, *n* = 62	38	61.3	47	75.8
Reported with measure of uncertainty, *n* = 38	19	50	27	57.5
Calibration, *n* = 62	3	4.8	11	17.7
Classification measures (e.g., sensitivity, specificity), *n* = 62	4	6.5	43	69.4
Clinical utility measures (e.g., decision curve analysis), *n* = 62	0	0	5	8.1
Is the potential clinical usefulness of the model(s) stated in the abstract or discussed in the main text? *n* = 62				
Yes	30	48.4	47	75.8
No	32	51.6	15	34.2
What is the recommended next step for the prediction model? *n* = 62				
To be used in clinical practice	12	19.4	11	17.7
Other recommendations for further study	6	9.7	23	37.1
Validate the models in a different setting/population	4	6.5	21	33.9
Unclear/not reported	40	64.5	7	11.3
Are the conclusions consistent with the reported study results? *n* = 62				
Yes	47	75.8	52	83.9
No	10	16.1	10	16.1
Conclusion section not included	5	8.1	-	-
Number of models to be assessed/developed *n* = 62				
Median (IQR), range	1 (1 to 3), 1 to 29		2 (1 to 4), 1 to 6	
Number reported (%)	46 (74.2%)		62 (100%)	

Values are numbers and percentages, unless otherwise specified.

a Values do not add to 100%, each are out of 62 studies.

Discrimination measures (such as the AUC and c-index) were the most commonly reported model performance measure in the abstract results section (*n* = 38/62, 55%), however, only half reported confidence intervals (*n* = 19/38, 50%). Classification measures (e.g., sensitivity, specificity, accuracy) were reported in four study abstracts (*n* = 4/62, 6%), calibrations measures were only reported in three study abstracts (*n* = 3/62, 5%), and no study reported clinical utility measure (e.g., decision curve analysis) in the abstract.

Almost half of the studies made statements about the potential clinical usefulness of the developed models (*n* = 30/62, 48%). However, over two-thirds did not clearly report the recommended next step for the developed prediction models (*n* = 40/62, 65%) in the abstract, compared to seven studies that were unclear about the next steps in the main text (*n* = 7/62, 11%). In the main text, most studies recommended the next step for the developed models to be validation in different settings or populations, including one study specifying the need for a larger dataset [36]. However, 11 studies (*n* = 11/62, 18%) indicated next steps for the prediction models should be used in clinical practice (six development-only studies and five development with validation studies), of which none evaluated clinical utility and only three studies evaluated calibration (using a calibration plot, slope, intercept, or table) [57,65,66]. For 22 studies (*n* = 22/62, 35%) other recommendations for further study were made, including use of larger data [43] and implementation of the model in a user-friendly application [35].

### 3.5 Use of leading or strong words

Thirty-five studies (*n* = 35/62, 56%) inappropriately or unjustifiably used a strong or leading word in their title, abstract, results, discussion, or conclusion, of which half inappropriately used strong or leading word in the abstract (*n* = 17/35, 49%), including words such as ‘novel’, ‘accurate,’ and ‘superior’ (Table 4). These words were used inappropriately as methodological robustness, results, and strength of evidence did not support their use. Example statements of the use of strong or leading words are presented in Box 1 and included “ensemble methods provided substantial advantages over single-model methods across all outcomes,” a generalization not supported by evidence in the abstract results [69] and “the ANN outperforms logistic regression, suggesting the importance of inter-factor coupling”, a statement supported by only discrimination and not considering calibration in the main text conclusion [35].

**Table 4:** Strong and leading words used in the title, abstract, results, discussion, and conclusion

Paper section	No of studies (%)	Strong or leading words used inappropriately^a^								
		Novel	Excellent	Accurate	Optimal	Powerful	Effective	Superior	Outperform	Other^b^
Title	4 studies, 6%	1	-	1	-		-	-	-	2
Abstract	17 studies, 27%	4	-	7	-	1	3	3	2	3
Results	15 studies, 24%	-	2	-	2		-	3	4	5
Discussion	14 studies, 23%	2	-	1	-	1	2	2	2	8
Conclusion	9 studies, 15%	-	-	3		1	1	1	2	5

a Use of these words were considered inappropriate as methodological robustness, results and strength of evidence did not support their use.

b Other words for title (“accuracy enhanced” and “new”), abstract (“favourably high”, “substantial advantages”, “reliably”), results (“susceptible”, “well known, reputable” and “confidently”, “perfect”, “significant”), discussion (“satisfactory and credible”, “precise”, “markedly better”, “much better”, “remarkable”, “milestone”, “efficient”, “markedly”) and conclusion (“appropriate”, “reliably”, “satisfactory”, “consistence” and “successful”).

Box 1 **Example statements of the use of strong and leading words**

Title (*n* = 4 studies)

“Accuracy Enhanced Lung Cancer Prognosis for Improving Patient Survivability Using Proposed Gaussian Classifier System” [67].
“Age and Lymphovascular Invasion Accurately Predict Sentinel Lymph Node Metastasis in T2 Melanoma Patients” [41].
“A novel prediction method for lymph node involvement in endometrial cancer: machine learning” [68].

Abstract (*n* = 17 studies)

“Using gradient boosting machine learning algorithms, it was possible to create a prediction model superior to conventional statistical methods” [51].
“Statistics can provide inferences within an overall system, while ML is a novel methodology that can make predictions” [53].
“Ensemble methods provided substantial advantages over single-model methods for all outcomes.” [69].

Results (*n* = 15 studies)

“The data illustrate that the tree ensembles Random Forest and RUSBoost display superior performance than single trees” [70].
“the optimal prediction model for the test set was the model constructed using the random forest classifier” [64].
“the optimal technique for the prediction of leukopenia was RF.” [71].

Discussion (*n* = 14 studies)

“In this second pilot study, our analysis demonstrated that a deep-learning neural network model is superior to conventional linear regression modeling in survival prediction for women with newly diagnosed cervical cancer” [36].
“the authors showed that their machine-learning models were markedly better calibrated than conventional statistical modelling” [42].
“highlight the power of the machine learning techniques for future studies.” [70].

Conclusion (*n* = 9 studies)

“The ANN outperforms logistic regression, suggesting the importance of inter-factor coupling” [35].
“ML is a powerful, albeit underutilized, tool in clinical medicine with direct relevance to neurosurgical outcomes research.” [72].
“In conclusion, an easy-to-use decision tree model for predicting the prognosis of individual patients with spinal metastasis was established with a satisfactory accuracy and consistence.” [65].

Spin was also indicated in the receiver operating characteristics curves and calibration plots from three studies. All three studies squashed the axes on their figures [65,67,73] and one study also truncated the axes [65].

## 4 Discussion

### 4.1 Summary of results

We reviewed 62 studies, described areas highly suggestive of spin and evaluated the possible presence of spin adapting and using existing spin frameworks and guidance. We found many inconsistencies in the reporting of key information between the methods, results, discussion, and conclusion section of the main text and also between the abstract and main text of the included studies. Key inconsistencies were between the analyses specified in the methods section and the findings reported in the results section of the main text. Such inconsistency could be viewed as spinning or overemphasizing study findings that may have not been planned or downplaying findings from analyses that were planned. Contributing to this inconsistency is the unavailability of a published or unpublished protocol for all included studies. The most common inconsistency was the addition of unplanned analyses in the results, followed by selective reporting of results from analyses specified in the methods.

We also found differences in the reporting of the study findings between the abstract and the main text. Most notably, in the study abstracts, up to 29 models were indicated to have been developed in any one single study, this compares to only up to six models that were reported to have been developed in the main text. This may be due to limited abstract word counts; however, this would not preclude studies from accurately reporting the total number of models to be developed in their study.

Model performance measures were less reported in the abstract compared to the main text and fewer were supported with measures of uncertainty. When reporting model performance measures, discrimination was emphasized considerably more than calibration, which was poorly reported, a finding that was consistent between the abstract and main text of studies. Discrimination is arguably an ‘easy’ measure to maximize or inflate to give the impression of good model performance–it is also bounded by 1 (perfect discrimination) and is thus relatively straight forward to interpret. It is also a rank order statistic for predictions against the observed outcome–such that adding 0.1 to all the estimated probabilities will lead to a model that systematically over predicts but will keep the same rank ordering and so discrimination will remain the same [74]. Whereas, demonstrating good calibration is a harder task, as it requires showing that the estimated probabilities from the model agree with what was observed and a larger sample size to reliably estimate [75,76].

Misleading reporting was the most prevalent domain of spin in the included studies and was compounded by the inappropriate and unjustified use of strong and leading words to interpret study findings with use of words, such as ‘superior’ and ‘outperforms,’ when comparing model performance. We found that these terms were especially used when making comparisons between nonregression (e.g., neural networks) and regression machine learning methods (e.g., logistic regression), which were often unfair and led to conclusions that nonregression machine learning methods were better than regression-based machine learning methods. For example, studies would often compare complex machine learning models that implicitly model nonlinearity and interactions to ‘standard’ logistic regression models, where nonlinearity or interactions would not be explored. Unfair comparisons between developed prediction models, in particular between machine learning and regression models, is an issue for prediction model research and can be a result of researcher bias.

Modern prediction model research is carried out in the backdrop of a surging interest in applying machine learning methods that is often perceived as being driven more by the aforementioned hype [77]. In the current climate, developing and validating machine learning prediction models can also be viewed as an enthusiasm in emerging research and technology arguably to progress careers and generate additional funding for scientific research, over contributing relevant, robust, applicable, meaningful, and needed research [78].

Naming a study as machine learning, could itself be considered spin, especially when portrayed in a favourable light. In the current ‘topical’ climate machine learning studies can increase chances of publication and garner readers more now than, say, 5 years ago. This may also explain why prediction model studies using logistic regression are now often branded as machine learning. Including machine learning and artificial intelligence in research profiles may also help researchers gain funding and facilitate career progression. Indeed, the use of promotional language can also help engage readers and convince other researchers/policy makers/funders that their research has been worthwhile and should be funded/adopted but can be at odds with ensuring research is not inappropriately ‘spun.’ Given the large amount of researcher and financial investment into artificial intelligence and machine learning, risk of bias from needing to show success and return and can lead (has led) to unfair comparisons between models, which can be gamed comparisons to ensure positive results.

### 4.2 Current literature

There is limited evidence regarding spin in prediction model research and even less evidence is available for machine learning prediction model research. Our study is the first study to address the latter limitation for prediction models in oncology. We can however draw parallels with a systematic review of prognostic factor studies in oncology [30] which also found selective and incomplete reporting, use of linguistic spin in the form of statements using strong and leading words and inconsistencies between the study methods and results and also between the study main text and abstract. Kempf et al. found inconsistency between the main text and abstract from 16 prognostic factor studies in oncology [30], which was much higher in our review of prediction modeling studies in oncology.

Several studies have also now highlighted the concern regarding the completeness of reporting, the risk of bias, and methodological conduct of studies using machine learning when developing clinical prediction models [16,[79], [80], [81]]. Overinterpreting model performance in the presence of poor or incorrect methodology and incomplete or poor reporting only further exacerbates concerns–creating research waste, and potential harm if implemented with insufficient robust evidence to support their use.

### 4.3 Strengths and limitations

Our study is the first study to review and evaluate spin in studies developing and validating prediction modeling research that use machine learning methods in oncology. Our study was limited however in adapting existing and available frameworks and guidance to detect spin from randomised controlled trials and prognostic factor studies, rather than using a bespoke prediction modeling framework. We may therefore have missed other important areas of spin, which would most likely support our conclusions. We used existing and available frameworks in these other areas to help inform our data extraction form and items to detect spin in the included studies. We also adopted a more descriptive approach when reviewing studies to highlight areas highly suggestive of spin rather than to explicitly classifying it in prediction model studies.

In relation to spin when comparing prediction models, we focused on assessing possible spin in the discussion section and did not assess the results section, which we assumed would be more objectively written and without interpretation. In doing this, we may have missed possible spin practice in the results section, which only further emphasize the spin present in prediction model studies with machine learning. We could not use study registration or study protocols to assess any prespecified methods, as these were not reported. We therefore assessed reporting consistency between the study methods and results.

Our search string was developed and executed in 2019 and searched for an approximately 8-month time period. Our search may be considered both short and outdated. However, we aimed to review a sample of papers to reflect current practice, as a more comprehensive review was not feasible given the very large number of prediction models that are being developed in oncology alone. At the time the search was run, it provided a contemporary sample of machine learning prediction models in oncology, which reflected and arguably still reflects the current status of prediction modeling research. Further, our study is an initial step in the evaluation of spin in these modeling studies. There have been no initiatives to improve the reporting of machine learning studies or strategies to minimize spin in these studies, and thus there is no expectation that in taking a more contemporary cohort that things will have improved. Conversely, we expect spin practice will have got worse over time given the number of publications that have arisen due to the pandemic. We have limited our review on spin practice in machine learning studies. This was not to further separate disparities between machine learning and statistical modeling methods, indeed, we may expect to find similar findings in studies not declaring their studies as using machine learning methods. Instead, we highlight spin practice and its possible influence in an area where there has been much hype but little gain.

### 4.4 Future research

Researchers developing and validating prediction models are required to plan and appropriately report findings for their studies. We provide some recommendations in Box 2 to avoid spin in prediction model research. Though current guidance for prediction model studies using regression-based models is available and also applicable to studies using machine learning models, bespoke guidance is needed for machine learning prediction model research that also captures the nuances in study design, analysis, and reporting for these studies. TRIPOD-AI is currently under development, which will provide researchers with the minimum key items for reporting their studies adequately [20]. Based on the findings of this review regarding linguistic spin, consideration also needs to be given to the language used when reporting study results and conclusions to avoid statements using strong or leading words, creating further hype for machine learning.

Box 2 **Recommendations to reduce spin in prediction model research**

1. Register the study and make the protocol available
  • Register your study on platforms like clinicaltrials.gov or the Open Science Framework (www.osf.io) so that prespecified comparisons with the main results paper can be made
  • Develop a protocol and make it publicly available either in a peer-reviewed journal, preprint server (e.g., medRxiv), or make it available on platforms like the Open Science Framework, where additional study documentation can be uploaded (e.g., statistical analysis plans, analytical code, and data) [82].
2. Ensure comparisons between models are preplanned, fair, and accounted for in the study design
  • Prespecify the aim and methods (e.g., in the protocol, statistical analysis plan, or registry) to compare any models, either comparing between developed models or comparing developed models to previously published models
  • Take steps to minimize researcher bias [83]. Ensure all models are fairly developed with the same level of objective flexibility to meet the goals of the intended use of the prediction model
  • Avoid meaningless comparisons of complex and flexible models against ‘vanilla’ models where additional flexibility has not been explored [84]. For example, avoid comparing a complex convolutional neural network to a ‘standard’ logistic regression where additional flexibility, such as restricted cubic splines or fractional polynomials, has not been explored to address nonlinearity
  • Ensure the data used to externally validate (‘test’) any models have not been used in their development
  • Consider the sample size requirements to conclude differences in performance between models
  • Do not rely on a single-model performance measure in isolation (e.g., do not solely consider discrimination and overlook calibration [85] or clinical utility [86]). Report multiple performance measures (e.g., discrimination and calibration as a minimum), when comparing models
3. Ensure the study abstract accurately reflects the findings reported in the main text (and those specified in the protocol)
  • An abstract extension to TRIPOD (transparent reporting of multivariable prediction models in journal and conference abstracts: TRIPOD for abstracts) is available to ensure prediction model study abstracts are reported more completely [87].
4. Use reporting guidelines to ensure the minimum required information is reported for the study
  • The TRIPOD statement is the recommended reporting guideline for studies describing the development or validation of a clinical prediction model [31,32].
5. Review language and ensure claims are supported by the study findings and accounts for strength of evidence
  • Avoid sensationalist language and the use of unnecessary adjectives that place an overly positive description of findings

Additional guidance is also needed for protocol development of prediction model studies to ensure analyses are robust, appropriate for the research question, and preplanned to avoid the additional analyses and selective reporting. Guidance is currently underway to provide reporting recommendations for protocols of prediction model research. Irrespective of the availability a formal reporting guideline for protocols, we strongly encourage researchers to have and make available, a protocol for their prediction model studies to ensure all facets of the study design have been considered and the issues identified in this review can be reduced.

A framework is also needed to formally assess the spin framework in prediction model research. This framework should consider misleading reporting, interpretation, and extrapolation as areas of priorities for spin assessment.

### 4.5 Conclusions

Caution is needed when reading, interpreting, and using studies that developed prediction models for cancer. Future prediction modeling research studies should develop and make study protocols available to ensure preplanned analyses and more robust and accurately reported studies. Researchers need to ensure any comparison between developed models and between developed and existing published models is fair by fully reporting methods, reporting results within context of possible differences between development and validation studies and datasets. Researchers should also be careful in their choice of words when reporting their study results and conclusions which should better reflect the strength of evidence produced by their study.
