# Supplementary material for: Overinterpretation of findings in machine learning prediction model studies in oncology: a systematic review
Source: J Clin Epidemiol. 2023 May;157:120–33. doi: 10.1016/j.jclinepi.2023.03.012 (PMC11913775; doi:10.1016/j.jclinepi.2023.03.012)
Supplement: Supplementary Tables [file mmc1.docx]

**Supplementary information**

**Supplementary table 1 - MEDLINE search strategy**

Database and platform: MEDLINE (Ovid MEDLINE® Epub Ahead of Print, In-Process & Other Non-Indexed Citations, Ovid MEDLINE® Daily and Ovid MEDLINE®) 1946 to present (via Ovid)

Publication date limit: 2019 only

Search date: 5 September 2019

| 1 | Machine Learning/ |
| --- | --- |
| 2 | (machine adj1 (learn$ or model$)).ti,ab,kw. |
| 3 | Deep Learning/ |
| 4 | (deep adj2 learn$).ti,ab,kw. |
| 5 | exp Supervised Machine Learning/ |
| 6 | (supervised adj2 machine adj2 learn$).ti,ab,kw. |
| 7 | ((support or relevance) adj2 vector adj2 (machine$ or classification$)).ti,ab,kw. |
| 8 | "Neural Networks (Computer)"/ |
| 9 | (neural adj2 network$).ti,ab,kw. |
| 10 | ((statistical or "statistical-learning") adj1 (learn$ or strateg$)).ti,ab.kw. |
| 11 | (multi adj2 layer adj1 perceptron$).ti,ab,kw. |
| 12 | (random adj2 forest$).ti,ab,kw. |
| 13 | "RF classifi$".ti,ab,kw. |
| 14 | (lasso or ridge or kernel or ensemble or bagging or bagged or bootstrap$ or boosting or boosted or fuzzy).ti,ab,kw. |
| 15 | ((penali?ed or regulari?ed) adj2 ('likelihood' or 'regression' or 'logistic' or 'survival' or 'estimat$' or 'function$' or 'method$' or 'least' or 'ensemble')).ti,ab,kw. |
| 16 | ((classification or regression or estimation or decision) adj2 tree$).ti,ab,kw. |
| 17 | (bayes$ adj1 network$).ti,ab,kw. |
| 18 | (nearest adj1 neighbo?r).ti,ab,kw. |
| 19 | (k-nearest adj1 neighbo?r).ti,ab,kw. |
| 20 | (elastic adj1 net).ti,ab,kw. |
| 21 | (naive adj1 bayes$).ti,ab,kw. |
| 22 | ((nonparametric or "non-parametric") adj2 (model$ or analys$)).ti,ab,kw |
| 23 | (KNN or ANN or ANNs or RNN or RF or SVM or NB or CART or DT or MLP).ti,ab,kw. |
| 24 | or/1-23 |
| 25 | Logistic Models/ |
| 26 | (logistic adj2 (model$ or regression)).ti,ab,kw. |
| 27 | Linear Models/ |
| 28 | (linear adj2 (model$ or regression)).ti,ab,kw. |
| 29 | (proportion$ adj2 odds adj2 regression).ti,ab,kw. |
| 30 | Least-Squares Analysis/ |
| 31 | (least adj2 square$).ti,ab,kw. |
| 32 | Survival Analysis/ |
| 33 | (survival adj1 (analys$ or model$)).ti,ab,kw. |
| 34 | Proportional Hazards Models/ |
| 35 | (proportional adj1 hazard$).ti,ab,kw. |
| 36 | ((cox or parametric) adj1 (regression or model$)).ti,ab,kw. |
| 37 | (semi adj2 parametric adj1 (regression or model$)).ti,ab,kw. |
| 38 | Disease-Free Survival/ |
| 39 | Progression-Free Survival/ |
| 40 | ((disease or progression or event) adj2 free adj1 survival).ti,ab,kw. |
| 41 | (overall adj1 survival).ti,ab,kw. |
| 42 | or/25-41 |
| 43 | 24 and 42 |
| 44 | 24 or 43 |
| 45 | exp Neoplasms/ |
| 46 | (cancer$ or tumour$ or tumor$ or carcinoma$ or malignan$ or neoplas$ or sarcoma$ or adenocarcinoma$ or carcinogen$ or metasta$ or oncolog$).ti,ab,kw. |
| 47 | or/45-46 |
| 48 | Prognosis/ |
| 49 | (prognos$ adj1 (modelling or modeling or model or models or predict$ or index or performance or nomogram or tools or ability or accuracy or probability or risk or factor$ or marker$ or biomarker$)).ti,ab,kw. |
| 50 | "risk model$".ti,ab,kw. |
| 51 | "predict$ the prognosis of".ti,ab,kw. |
| 52 | "predict$ the risk of".ti,ab,kw. |
| 53 | "predict$ the probability of".ti,ab,kw. |
| 54 | Probability/ |
| 55 | (probability adj1 (modelling or modeling or model or models)).ti,ab,kw. |
| 56 | (predict$ adj1 (modelling or modeling or model or models or nomogram or tools or performance or ability or index or accuracy or probability or risk or factor$ or marker$ or biomarker$)).ti,ab,kw. |
| 57 | "candidate predictor$".ti,ab,kw. |
| 58 | "predictive clinical parameter$".ti,ab,kw. |
| 59 | ((discrimination or discriminative or discriminatory) adj1 (accuracy or ability or performance or value or model or models or power or capacity or capabilit$ or efficiency)).ti,ab,kw. |
| 60 | (discriminability or c-index or c-statistic or concordance or DCA).ti,ab,kw. |
| 61 | "decision curve".ti,ab,kw. |
| 62 | (calibrat$ adj1 (plot$ or curve$ or slope$ or model or models)).ti,ab,kw. |
| 63 | (brier adj1 score$).ti,ab,kw. |
| 64 | (performance adj1 (classification or classifier or clinical or accuracy or validation or metrics or diagnostic or AUC)).ti,ab,kw. |
| 65 | (sensitivity or specificity or PPV or NPV).ti,ab,kw. |
| 66 | "correctly classified".ti,ab,kw. |
| 67 | "clinical accuracy".ti,ab,kw. |
| 68 | "positive predictive value$".ti,ab,kw. |
| 69 | "negative predictive value$".ti,ab,kw. |
| 70 | (classification or classifier).ti,ab,kw. |
| 71 | Area Under Curve/ |
| 72 | "Area under the curve".ti,ab,kw. |
| 73 | "Area under the ROC curve".ti,ab,kw. |
| 74 | "Area under the ROC".ti,ab,kw. |
| 75 | "Area Under the Receiver Operat$ Characteristic$".ti,ab,kw. |
| 76 | ROC Curve/ |
| 77 | "receiver operating characteristic$".ti,ab,kw. |
| 78 | (ROC or AUC or AUROC).ti,ab,kw. |
| 79 | "Hosmer-Lemeshow".ti,ab,kw. |
| 80 | "H-L test".ti,ab,kw. |
| 81 | "expected ratio".ti,ab,kw. |
| 82 | "observed ratio".ti,ab,kw. |
| 83 | "E:O ratio".ti,ab,kw. |
| 84 | or/48-83 |
| 85 | 44 and 47 and 84 |
| 86 | Limit 85 to yr="2019" |

**Supplementary table 2 - EMBASE search strategy**

Database and platform: Embase 1974 to present (via Ovid)

Publication d limit: 2019 only

Search date: 5 September 2019

| 1 | exp Machine Learning/ |
| --- | --- |
| 2 | (machine adj1 (learn$ or model$)).ti,ab,kw. |
| 3 | (deep adj2 learn$).ti,ab,kw. |
| 4 | (supervised adj2 machine adj2 learn$).ti,ab,kw. |
| 5 | ((support or relevance) adj2 vector adj2 (machine$ or classification$)).ti,ab,kw. |
| 6 | (neural adj2 network$).ti,ab,kw. |
| 7 | ((statistical or "statistical-learning") adj1 (learn$ or strateg$)).ti,ab,kw. |
| 8 | (multi adj2 layer adj1 perceptron$).ti,ab,kw. |
| 9 | (random adj2 forest$).ti,ab,kw. |
| 10 | "RF classifi$".ti,ab,kw. |
| 11 | Bootstrapping/ |
| 12 | (lasso or ridge or kernel or ensemble or bagging or bagged or bootstrap$ or boosting or boosted or fuzzy).ti,ab,kw. |
| 13 | ((penali?ed or regulari?ed) adj2 (likelihood or regression or logistic or survival or estimat$ or function$ or method$ or least or ensemble)).ti,ab,kw. |
| 14 | Decision Tree/ |
| 15 | ((classification or regression or estimation or decision) adj2 tree$).ti,ab,kw. |
| 16 | (naive adj1 bayes$).ti,ab,kw. |
| 17 | (bayes$ adj1 network$).ti,ab,kw. |
| 18 | (nearest adj1 neighbo?r).ti,ab,kw. |
| 19 | (k-nearest adj1 neighbo?r).ti,ab,kw. |
| 20 | (elastic adj1 net).ti,ab,kw. |
| 21 | Nonparametric Test/ |
| 22 | ((nonparametric or "non-parametric") adj2 (model$ or analys$)).ti,ab,kw. |
| 23 | (KNN or ANN or ANNs or RNN or RF or SVM or NB or CART or DT or MLP).ti,ab,kw. |
| 24 | or/1-23 |
| 25 | Logistic Regression Analysis/ |
| 26 | (logistic adj2 (model$ or regression)).ti,ab,kw. |
| 27 | Linear Regression Analysis/ |
| 28 | (linear adj2 (model$ or regression)).ti,ab,kw. |
| 29 | (proportion$ adj2 odds adj2 regression).ti,ab,kw. |
| 30 | Least Square Analysis/ |
| 31 | (least adj2 square$).ti,ab,kw. |
| 32 | Survival Analysis/ |
| 33 | (survival adj1 (analys$ or model$)).ti,ab,kw. |
| 34 | Proportional Hazards Models/ |
| 35 | (proportional adj1 hazard$).ti,ab,kw. |
| 36 | ((cox or parametric) adj1 (regression or model$)).ti,ab,kw. |
| 37 | (semi adj2 parametric adj1 (regression or model$)).ti,ab,kw. |
| 38 | Disease-Free Survival/ |
| 39 | Progression-Free Survival/ |
| 40 | ((disease or progression or event) adj2 free adj1 survival).ti,ab,kw. |
| 41 | (overall adj1 survival).ti,ab,kw. |
| 42 | or/25-41 |
| 43 | 24 and 42 |
| 44 | 24 or 43 |
| 45 | exp Neoplasm/ |
| 46 | (cancer$ or tumour$ or tumor$ or carcinoma$ or malignan$ or neoplas$ or sarcoma$ or adenocarcinoma$ or carcinogen$ or metasta$ or oncolog$).ti,ab,kw. |
| 47 | or/45-46 |
| 48 | exp Prognosis/ |
| 49 | (prognos$ adj1 (modelling or modeling or model or models or predict$ or index or performance or nomogram or tools or ability or accuracy or probability or risk or factor$ or marker$ or biomarker$ or parameter$)).ti,ab,kw. |
| 50 | "risk model$".ti,ab,kw. |
| 51 | "predict$ the prognosis of".ti,ab,kw. |
| 52 | "predict$ the risk of".ti,ab,kw. |
| 53 | "predict$ the probability of".ti,ab,kw. |
| 54 | (probability adj1 (modelling or modeling or model or models)).ti,ab,kw. |
| 55 | Prediction/ |
| 56 | (predict$ adj1 (modelling or modeling or model or models or nomogram or tools or performance or ability or index or accuracy or probability or risk or factor$ or marker$ or biomarker$)).ti,ab,kw. |
| 57 | "candidate predictor$".ti,ab,kw. |
| 58 | "predictive clinical parameter$".ti,ab,kw. |
| 59 | ((discrimination or discriminative or discriminatory) adj1 (accuracy or ability or performance or value or model or models or power or capacity or capabilit$ or efficiency)).ti,ab,kw. |
| 60 | (discriminability or c-index or c-statistic or concordance or DCA).ti,ab,kw. |
| 61 | "decision curve".ti,ab,kw. |
| 62 | Calibration/ |
| 63 | (calibrat$ adj1 (plot$ or curve$ or slope$ or model or models)).ti,ab,kw. |
| 64 | (brier adj1 score$).ti,ab,kw. |
| 65 | (performance adj1 (classification or classifier or clinical or accuracy or validation or metrics or diagnostic or AUC)).ti,ab,kw. |
| 66 | Validation Process/ |
| 67 | (sensitivity or specificity or PPV or NPV).ti,ab,kw. |
| 68 | "correctly classified".ti,ab,kw. |
| 69 | (classification or classifier).ti,ab,kw. |
| 70 | "clinical accuracy".ti,ab,kw. |
| 71 | "positive predictive value$".ti,ab,kw. |
| 72 | "negative predictive value$".ti,ab,kw. |
| 73 | Predictive value/ |
| 74 | Probability/ |
| 75 | "Area Under the Curve"/ |
| 76 | "Area Under the Curve Ratio"/ |
| 77 | "Area under the curve".ti,ab,kw. |
| 78 | "Area under the ROC curve".ti,ab,kw. |
| 79 | "Area under the ROC".ti,ab,kw. |
| 80 | "Area Under the Receiver Operat$ Characteristic$".ti,ab,kw. |
| 81 | ROC Curve/ |
| 82 | Receiver Operating Characteristic/ |
| 83 | "receiver operating characteristic$".ti,ab,kw. |
| 84 | (ROC or AUC or AUROC).ti,ab,kw. |
| 85 | "Hosmer-Lemeshow".ti,ab,kw. |
| 86 | "H-L test".ti,ab,kw. |
| 87 | "expected ratio".ti,ab,kw. |
| 88 | "observed ratio".ti,ab,kw. |
| 89 | "E:O ratio".ti,ab,kw. |
| 90 | or/48-89 |
| 91 | 44 and 47 and 90 |
| 92 | conference abstract.pt. |
| 93 | conference abstract.st. |
| 94 | 92 or 93 |
| 95 | 91 not 94 |
| 96 | Limit 95 to yr="2019" |

**Supplementary table 3 – extracted spin items**

| Is adherence to a reporting guideline mentioned? |
| --- |
| If yes, what reporting guideline was used? |
| If other, please specify |
| Was a protocol for the study referenced? |
| Is there any subgroup analysis specified? |
| If yes, how many? |
| Is there any sensitivity analysis specified? |
| If yes, how many? |
| Are results consistent with what was prespecified? |
| If not, why? |
| Is reporting of results of subgroup analysis consistent with what was prespecified? |
| Is reporting of results of sensitivity analysis consistent with what was prespecified? |
| Is there any spin in presentation of figures (calibration/ROC)? |
| If yes, please specify (choice=Plot truncated) |
| If yes, please specify (choice=Squashed axes) |
| If yes, please specify (choice=Other) |
| If other, please specify |
| Consistent use of categorisation of continuous predictors? |
| Any indication of misleading strategy on how continuous data was treated? |
| If yes, please specify |
| Is there use of leading words/strong statement in the results to describe model and/or model performance? |
| If so, please specify the leading word/strong statement (choice=Novel) |
| If so, please specify the leading word/strong statement (choice=Excellent) |
| If so, please specify the leading word/strong statement (choice=Accurate) |
| If so, please specify the leading word/strong statement (choice=Optimal) |
| If so, please specify the leading word/strong statement (choice=Perfect) |
| If so, please specify the leading word/strong statement (choice=Significant) |
| If so, please specify the leading word/strong statement (choice=Other) |
| If other, please specify |
| Please provide examples of the spin statements found |
| Are any other misleading strategies reported in the results? |
| If yes, please specify |
| If yes, what is the funding source |
| Does journal have conflicts of interest section? |
| How many authors declared COI? |
| Are comparisons made between the developed models in the present study? |
| What was the final claim made from the comparison? |
| What results were used to support this claim? |
| Is there a reference to a previously published model? |
| If yes, is the developed model better or worse than the published model? |
| Did authors give reasons for the difference in the performance? |
| Did authors compare machine learning to non-machine learning models? |
| Was machine learning declared better than non-machine learning? |
| Is there an emphasis on model relevance while results are not predictive? |
| What is the recommended next step for the prediction model? |
| If other, please specify |
| Is there use of leading words/strong statement in the discussion to describe model and/or model performance? |
| If so, please specify the leading word/strong statement (choice=Novel) |
| If so, please specify the leading word/strong statement (choice=Excellent) |
| If so, please specify the leading word/strong statement (choice=Accurate) |
| If so, please specify the leading word/strong statement (choice=Optimal) |
| If so, please specify the leading word/strong statement (choice=Perfect) |
| If so, please specify the leading word/strong statement (choice=Significant) |
| If so, please specify the leading word/strong statement (choice=Other) |
| If other, please specify |
| Please provide examples of the spin statements found |
| Are any other misleading strategies reported in the discussion? |
| If yes, please specify |
| Are conclusions consistent with the reported results? |
| Is there use of leading words/strong statement in the conclusion to describe model and/or model performance? |
| If so, please specify the leading word/strong statement (choice=Novel) |
| If so, please specify the leading word/strong statement (choice=Excellent) |
| If so, please specify the leading word/strong statement (choice=Accurate) |
| If so, please specify the leading word/strong statement (choice=Optimal) |
| If so, please specify the leading word/strong statement (choice=Perfect) |
| If so, please specify the leading word/strong statement (choice=Significant) |
| If so, please specify the leading word/strong statement (choice=Other) |
| If other, please specify |
| Please provide examples of the spin statements found |
| Are any other misleading strategies reported in the conclusion? |
| If yes, please specify |
| Is there use of leading words/strong statement in the title to describe model and/or model performance? |
| If so, please specify the leading word/strong statement (choice=Novel) |
| If so, please specify the leading word/strong statement (choice=Excellent) |
| If so, please specify the leading word/strong statement (choice=Accurate) |
| If so, please specify the leading word/strong statement (choice=Optimal) |
| If so, please specify the leading word/strong statement (choice=Perfect) |
| If so, please specify the leading word/strong statement (choice=Significant) |
| If so, please specify the leading word/strong statement (choice=Other) |
| If other, please specify |
| Please provide examples of the spin statements found |
| If yes, which measures were reported? |
| If yes, with precision estimates? |
| If yes, which measures were reported? |
| If yes, with precision estimates? |
| If yes, are the conclusions consistent with the reported study results? |
| Are statements about clinical usefulness made? |
| What is the recommended next step for the prediction model? |
| If the model is recommended for use in clinical practice, does the recommendation include using the model in a difference clinical setting/population? |
| If other, please specify |
| Is the number of models that will be assessed reported? |
| What is the reported number of models? |
| Is there a reference to compare the ML technique with traditional statistical methods? |
| Is there use of leading words/strong statement in the abstract (methods/results/conclusion) to describe model and/or model performance? |
| If so, please specify the leading word/strong statement (choice=Novel) |
| If so, please specify the leading word/strong statement (choice=Excellent) |
| If so, please specify the leading word/strong statement (choice=Accurate) |
| If so, please specify the leading word/strong statement (choice=Optimal) |
| If so, please specify the leading word/strong statement (choice=Perfect) |
| If so, please specify the leading word/strong statement (choice=Significant) |
| If so, please specify the leading word/strong statement (choice=Other) |
| If other, please specify |
| Please provide examples of the spin statements found |
| Are any other misleading strategies reported in the abstract? |
| If other, please specify |

**Supplementary table 4 – List of included studies**

| **Title** | **Study Design** |
| --- | --- |
| Accuracy Enhanced Lung Cancer Prognosis for Improving Patient Survivability Using Proposed Gaussian Classifier System [1] | Development only study |
| Analysis of survival for lung cancer resections cases with fuzzy and soft set theory in surgical decision making [2] | Development only study |
| Application of machine learning techniques to analyze anastomosis integrity after Total gastrectomy for prediction of clinical leakage [3] | Development only study |
| Artificial neural network models to predict nodal status in clinically node-negative breast cancer [4] | Development only study |
| askMUSIC: Leveraging a Clinical Registry to Develop a New Machine Learning Model to Inform Patients of Prostate Cancer Treatments Chosen by Similar Men [5] | Development only study |
| Assessment of Deep Learning Using Nonimaging Information and Sequential Medical Records to Develop a Prediction Model for Nonmelanoma Skin Cancer [6] | Development only study |
| Automated data extraction and ensemble methods for predictive modeling of breast cancer outcomes after radiation therapy [7] | Development only study |
| Can machine learning predict resecability of a peritoneal carcinomatosis? [8] | Development only study |
| Characteristics and long-term outcomes of advanced pleural mesothelioma in Latin America (MeSO-CLICaP) [9] | Development only study |
| Clinical characteristics and disease specific prognostic nomogram for primary gliosarcoma: a SEER population-based analysis [10] | Development only study |
| Creating Prognostic Systems for Well-Differentiated Thyroid Cancer Using Machine Learning [11] | Development only study |
| Deep learning-based survival prediction of oral cancer patients [12] | Development only study |
| Deep stacked sparse auto-encoders for prediction of postoperative survival expectancy in thoracic lung cancer surgery [13] | Development only study |
| Developing case-finding algorithms for second events of oropharyngeal cancer using administrative data: A population-based validation study [14] | Development only study |
| Development and Assessment of a Machine Learning Model to Help Predict Survival Among Patients With Oral Squamous Cell Carcinoma [15] | Development only study |
| Development and validation of case-finding algorithms for recurrence of breast cancer using routinely collected administrative data [16] | Development only study |
| Development of a Novel Prognostic Risk Score for Predicting Complications of Penectomy in the Surgical Management of Penile Cancer [17] | Development only study |
| Development of Deep Learning Algorithm for Detection of Colorectal Cancer in EHR Data [18] | Development only study |
| Development of Machine Learning Algorithms for Prediction of 30-Day Mortality After Surgery for Spinal Metastasis [19] | Development only study |
| Extent of Resection in Meningioma: Predictive Factors and Clinical Implications [20] | Development only study |
| Gait speed and survival of older surgical patient with cancer: Prediction after machine learning [21] | Development only study |
| Machine Learning Algorithm Identifies Patients at High Risk for Early Complications After Intracranial Tumor Surgery: Registry-Based Cohort Study [22] | Development only study |
| Machine learning methods applied to audit of surgical outcomes after treatment for cancer of the head and neck [23] | Development only study |
| Machine Learning to Predict Delays in Adjuvant Radiation following Surgery for Head and Neck Cancer [24] | Development only study |
| A machine-learning based prediction model of fistula formation after interstitial brachytherapy for locally advanced gynecological malignancies [25] | Development only study |
| A novel prediction method for lymph node involvement in endometrial cancer: machine learning [26] | Development only study |
| Posterior fossa meningiomas: perioperative predictors of extent of resection, overall survival and progression-free survival [27] | Development only study |
| Predicting 90-Day and 1-Year Mortality in Spinal Metastatic Disease: Development and Internal Validation [28] | Development only study |
| Predicting breast cancer metastasis by using serum biomarkers and clinicopathological data with machine learning technologies [29] | Development only study |
| Predictors of the therapeutic effect of corticosteroids on radiation-induced optic neuropathy following nasopharyngeal carcinoma [30] | Development only study |
| Predictive model algorithms identifying early and advanced stage ER+/HER2- breast cancer in claims data [31] | Development only study |
| Predicting cervical cancer screening among sexual minority women using Classification and Regression Tree analysis [32] | Development only study |
| Predicting Disease-Free Lung Cancer Survival Using Patient Reported Outcome (PRO) Measurements with Comparisons of Five Machine Learning Techniques (MLT) [33] | Development only study |
| Prediction of 10-year Overall Survival in Patients with Operable Cervical Cancer using a Probabilistic Neural Network [34] | Development only study |
| Predicting radiation pneumonitis in locally advanced stage II-III non-small cell lung cancer using machine learning [35] | Development only study |
| Predicting Survival of Patients with Spinal Ependymoma Using Machine Learning Algorithms with the SEER Database [36] | Development only study |
| Prediction of future gastric cancer risk using a machine learning algorithm and comprehensive medical check-up data: A case-control study [37] | Development only study |
| Prediction of irinotecan toxicity in metastatic colorectal cancer patients based on machine learning models with pharmacokinetic parameters [38] | Development only study |
| A Predictive Model for Postembolization Syndrome after Transarterial Hepatic Chemoembolization of Hepatocellular Carcinoma [39] | Development only study |
| A prospective study examining cachexia predictors in patients with incurable cancer [40] | Development only study |
| Using Machine Learning to Predict Progression in the Gastric Precancerous Process in a Population from a Developing Country Who Underwent a Gastroscopy for Dyspeptic Symptoms [41] | Development only study |
| Recursive Partitioning Analysis (RPA) of Prognostic Factors for Overall Survival in Patients with Spinal Metastasis: A New System for Stratified Treatment [42] | Development only study |
| Response to repeat echoendoscopic celiac plexus neurolysis in pancreatic cancer patients: A machine learning approach [43] | Development only study |
| Risk Factors for Local Relapse and Inferior Disease-free Survival After Breast-conserving Management of Breast Cancer: Recursive Partitioning Analysis of 2161 Patients [44] | Development only study |
| Survivability prediction of colon cancer patients using neural networks [45] | Development only study |
| Survival outcome prediction in cervical cancer: Cox models vs deep-learning model [46] | Development only study |
| Use of Machine Learning for Prediction of Patient Risk of Postoperative Complications After Liver, Pancreatic, and Colorectal Surgery [47] | Development only study |
| Use of machine learning to predict early biochemical recurrence after robot-assisted prostatectomy [48] | Development only study |
| Age and Lymphovascular Invasion Accurately Predict Sentinel Lymph Node Metastasis in T2 Melanoma Patients [49] | Development and  validation study |
| Early Warning Models to Estimate the 30-Day Mortality Risk After Stent Placement for Patients with Malignant Biliary Obstruction [50] | Development and  validation study |
| Machine learning application for prediction of locoregional recurrences in early oral tongue cancer: a Web-based prognostic tool [51] | Development and  validation study |
| Nomograms for predicting the overall and cause-specific survival in patients with malignant peripheral nerve sheath tumor: a population-based study [52] | Development and  validation study |
| Patient-based prediction algorithm of relapse after allo-HSCT for acute Leukemia and its usefulness in the decision-making process using a machine learning approach [53] | Development and  validation study |
| A predictive model of overall survival in patients with metastatic castration-resistant prostate cancer [54] | Development and  validation study |
| Predicting Inpatient Length of Stay After Brain Tumor Surgery: Developing Machine Learning Ensembles to Improve Predictive Performance [55] | Development and  validation study |
| Predicting Overall Survival in Patients with Metastatic Rectal Cancer: a Machine Learning Approach [56] | Development and  validation study |
| Prediction of survival outcomes in patients with epithelial ovarian cancer using machine learning methods [57] | Development and  validation study |
| Prediction of the 1-Year Risk of Incident Lung Cancer: Prospective Study Using Electronic Health Records from the State of Maine [58] | Development and  validation study |
| A Proposal to Reflect Survival Difference and Modify the Staging System for Lung Adenocarcinoma and Squamous Cell Carcinoma: Based on the Machine Learning [59] | Development and  validation study |
| Scoring colorectal cancer risk with an artificial neural network based on self-reportable personal health data [60] | Development and  validation study |
| Semi-supervised learning to improve generalizability of risk prediction models [61] | Development and  validation study |
| Use of Machine-Learning Algorithms in Intensified Preoperative Therapy of Pancreatic Cancer to Predict Individual Risk of Relapse [62] | Development and  validation study |

**References for supplementary tables**

1 Kaviarasi R, Gandhi RR . Accuracy Enhanced Lung Cancer Prognosis for Improving Patient Survivability Using Proposed Gaussian Classifier System. J Med Syst 2019;43:201. doi:10.1007/s10916-019-1297-2

2 Alcantud JCR, Varela G, Santos-Buitrago B, et al. Analysis of survival for lung cancer resections cases with fuzzy and soft set theory in surgical decision making. PLoS One 2019;14:e0218283. doi:10.1371/journal.pone.0218283

3 Celik S, Sohail A, Ashraf S, et al. Application of machine learning techniques to analyze anastomosis integrity after Total gastrectomy for prediction of clinical leakage. Health Technol 2019;9:757–63. doi:10.1007/s12553-019-00334-3

4 Dihge L, Ohlsson M, Edén P, et al. Artificial neural network models to predict nodal status in clinically node-negative breast cancer. BMC Cancer 2019;19:610. doi:10.1186/s12885-019-5827-6

5 Auffenberg GB, Ghani KR, Ramani S, et al. askMUSIC: Leveraging a Clinical Registry to Develop a New Machine Learning Model to Inform Patients of Prostate Cancer Treatments Chosen by Similar Men. Eur Urol 2019;75:901–7. doi:10.1016/j.eururo.2018.09.050

6 Wang H-H, Wang Y-H, Liang C-W, et al. Assessment of Deep Learning Using Nonimaging Information and Sequential Medical Records to Develop a Prediction Model for Nonmelanoma Skin Cancer. JAMA Dermatol 2019. 155(11):1277-1283. doi:10.1001/jamadermatol.2019.2335

7 Lindsay WD, Ahern CA, Tobias JS, et al. Automated data extraction and ensemble methods for predictive modeling of breast cancer outcomes after radiation therapy. Med Phys 2019;46:1054–63. doi:10.1002/mp.13314

8 Maubert A, Birtwisle L, Bernard JL, et al. Can machine learning predict resecability of a peritoneal carcinomatosis? Surg Oncol 2019;29:120–5. doi:10.1016/j.suronc.2019.04.008

9 Rojas L, Cardona AF, Trejo-Rosales R, et al. Characteristics and long-term outcomes of advanced pleural mesothelioma in Latin America (MeSO-CLICaP). Thorac Cancer 2019;10:508–18. doi:10.1111/1759-7714.12967

10 Feng S-S, Li H, Fan F, et al. Clinical characteristics and disease-specific prognostic nomogram for primary gliosarcoma: a SEER population-based analysis. Sci Rep 2019;9:10744. doi:10.1038/s41598-019-47211-7

11 Yang CQ, Gardiner L, Wang H, et al. Creating Prognostic Systems for Well-Differentiated Thyroid Cancer Using Machine Learning. Front Endocrinol (Lausanne) 2019;10:288. doi:10.3389/fendo.2019.00288

12 Kim DW, Lee S, Kwon S, et al. Deep learning-based survival prediction of oral cancer patients. Sci Rep 2019;9:6994. doi:10.1038/s41598-019-43372-7

13 Iraji MS. Deep stacked sparse auto-encoders for prediction of post-operative survival expectancy in thoracic lung cancer surgery. J Appl Biomed 2019;17:75–75. doi:10.32725/jab.2018.007

14 Xu Y, Kong S, Cheung WY, et al. Developing case-finding algorithms for second events of oropharyngeal cancer using administrative data: A population-based validation study. Head Neck 2019;41:2291–8. doi:10.1002/hed.25682

15 Karadaghy OA, Shew M, New J, et al. Development and Assessment of a Machine Learning Model to Help Predict Survival Among Patients With Oral Squamous Cell Carcinoma. JAMA Otolaryngol Head Neck Surg 2019;145(12):1115-1120. doi:10.1001/jamaoto.2019.0981

16 Xu Y, Kong S, Cheung WY, et al. Development and validation of case-finding algorithms for recurrence of breast cancer using routinely collected administrative data. BMC Cancer 2019;19:210. doi:10.1186/s12885-019-5432-8

17 Velazquez N, Press B, Renson A, et al. Development of a Novel Prognostic Risk Score for Predicting Complications of Penectomy in the Surgical Management of Penile Cancer. Clin Genitourin Cancer 2019;17:e123–9. doi:10.1016/j.clgc.2018.09.018

18 Wang Y-H, Nguyen P-A, Islam MM, et al. Development of Deep Learning Algorithm for Detection of Colorectal Cancer in EHR Data. Stud Health Technol Inform 2019;264:438–41. doi:10.3233/SHTI190259

19 Karhade AV, Thio QCBS, Ogink PT, et al. Development of Machine Learning Algorithms for Prediction of 30-Day Mortality After Surgery for Spinal Metastasis. Neurosurgery 2019;85:E83–91. doi:10.1093/neuros/nyy469

20 Lemée J-M, Corniola MV, Da Broi M, et al. Extent of Resection in Meningioma: Predictive Factors and Clinical Implications. Sci Rep 2019;9:5944. doi:10.1038/s41598-019-42451-z

21 Sasani K, Catanese HN, Ghods A, et al. Gait speed and survival of older surgical patient with cancer: Prediction after machine learning. J Geriatr Oncol 2019;10:120–5. doi:10.1016/j.jgo.2018.06.012

22 van Niftrik CHB, van der Wouden F, Staartjes VE, et al. Machine Learning Algorithm Identifies Patients at High Risk for Early Complications After Intracranial Tumor Surgery: Registry-Based Cohort Study. Neurosurgery 2019;85:E756–64. doi:10.1093/neuros/nyz145

23 Tighe D, Lewis-Morris T, Freitas A. Machine learning methods applied to audit of surgical outcomes after treatment for cancer of the head and neck. Br J Oral and Maxillofac Surg 2019;57:771–7. doi:10.1016/j.bjoms.2019.05.026

24 Shew M, New J, Bur AM. Machine Learning to Predict Delays in Adjuvant Radiation following Surgery for Head and Neck Cancer. Otolaryngol Head Neck Surg 2019;160:1058–64. doi:10.1177/0194599818823200

25 Tian Z, Yen A, Zhou Z, et al. A machine-learning–based prediction model of fistula formation after interstitial brachytherapy for locally advanced gynecological malignancies. Brachytherapy 2019;18:530–8. doi:10.1016/j.brachy.2019.04.004

26 Günakan E, Atan S, Haberal AN, et al. A novel prediction method for lymph node involvement in endometrial cancer: machine learning. Int J Gynecol Cancer 2019;29. doi:10.1136/ijgc-2018-000033

27 Corniola MV, Lemée J-M, Da Broi M, et al. Posterior fossa meningiomas: perioperative predictors of extent of resection, overall survival and progression-free survival. Acta Neurochir (Wien) 2019;161:1003–11. doi:10.1007/s00701-019-03862-z

28 Karhade AV, Thio QCBS, Ogink PT, et al. Predicting 90-Day and 1-Year Mortality in Spinal Metastatic Disease: Development and Internal Validation. Neurosurgery 2019;85:E671–81. doi:10.1093/neuros/nyz070

29 Tseng Y-J, Huang C-E, Wen C-N, et al. Predicting breast cancer metastasis by using serum biomarkers and clinicopathological data with machine learning technologies. Int J Med Inform 2019;128:79–86. doi:10.1016/j.ijmedinf.2019.05.003

30 Zheng B, Lin J, Li Y, et al. Predictors of the therapeutic effect of corticosteroids on radiation-induced optic neuropathy following nasopharyngeal carcinoma. Support Care Cancer 2019;27:4213–9. doi:10.1007/s00520-019-04699-z

31 Beachler DC, de Luise C, Yin R, et al. Predictive model algorithms identifying early and advanced stage ER+/HER2- breast cancer in claims data. Pharmacoepidemiol Drug Saf 2019;28:171–8. doi:10.1002/pds.4681

32 Greene MZ, Hughes TL, Hanlon A, et al. Predicting cervical cancer screening among sexual minority women using Classification and Regression Tree analysis. Prev Med Rep 2019;13:153–9. doi:10.1016/j.pmedr.2018.11.007

33 Sim J-A, Yun YH. Predicting Disease-Free Lung Cancer Survival Using Patient Reported Outcome (PRO) Measurements with Comparisons of Five Machine Learning Techniques (MLT). Stud Health Technol Inform 2019;264:1588–9. doi:10.3233/SHTI190548

34 Obrzut B, Kusy M, Semczuk A, et al. Prediction of 10-year Overall Survival in Patients with Operable Cervical Cancer using a Probabilistic Neural Network. J Cancer 2019;10:4189–95. doi:10.7150/jca.33945

35 Luna JM, Chao H-H, Diffenderfer ES, et al. Predicting radiation pneumonitis in locally advanced stage II-III non-small cell lung cancer using machine learning. Radiother Oncol 2019;133:106–12. doi:10.1016/j.radonc.2019.01.003

36 Ryu SM, Lee S-H, Kim E-S, et al. Predicting Survival of Patients with Spinal Ependymoma Using Machine Learning Algorithms with the SEER Database. World Neurosurg 2018; S1878-8750(18)32914-0. doi:10.1016/j.wneu.2018.12.091

37 Taninaga J, Nishiyama Y, Fujibayashi K, et al. Prediction of future gastric cancer risk using a machine learning algorithm and comprehensive medical check-up data: A case-control study. Sci Rep 2019;9:12384. doi:10.1038/s41598-019-48769-y

38 Oyaga-Iriarte E, Insausti A, Sayar O, et al. Prediction of irinotecan toxicity in metastatic colorectal cancer patients based on machine learning models with pharmacokinetic parameters. J Pharmacol Sci 2019;140:20–5. doi:10.1016/j.jphs.2019.03.004

39 Khalaf MH, Sundaram V, AbdelRazek Mohammed MA, et al. A Predictive Model for Postembolization Syndrome after Transarterial Hepatic Chemoembolization of Hepatocellular Carcinoma. Radiology 2019;290:254–61. doi:10.1148/radiol.2018180257

40 Vagnildhaug OM, Brunelli C, Hjermstad MJ, et al. A prospective study examining cachexia predictors in patients with incurable cancer. BMC Palliat Care 2019;18:46. doi:10.1186/s12904-019-0429-2

41 Thapa S, Fischback LA, Delongchamp R, et al. Using Machine Learning to Predict Progression in the Gastric Precancerous Process in a Population from a Developing Country Who Underwent a Gastroscopy for Dyspeptic Symptoms. Gastroenterol Res Pract 2019:8321942. doi:10.1155/2019/8321942.

42 Yang X-G, Wang F, Feng J-T, et al. Recursive Partitioning Analysis (RPA) of Prognostic Factors for Overall Survival in Patients with Spinal Metastasis: A New System for Stratified Treatment. World Neurosurg 2019;127:e124–31. doi:10.1016/j.wneu.2019.02.183

43 Facciorusso A, Del Prete V, Antonino M, et al. Response to repeat echoendoscopic celiac plexus neurolysis in pancreatic cancer patients: A machine learning approach. Pancreatology 2019;19:866–72. doi:10.1016/j.pan.2019.07.038

44 Hammer J, Geinitz H, Nieder C, et al. Risk Factors for Local Relapse and Inferior Disease-free Survival After Breast-conserving Management of Breast Cancer: Recursive Partitioning Analysis of 2161 Patients. Clin Breast Cancer 2019;19:58–62. doi:10.1016/j.clbc.2018.08.001

45 Al-Bahrani R, Agrawal A, Choudhary A. Survivability prediction of colon cancer patients using neural networks. Health Informatics J 2019;25:878–91. doi:10.1177/1460458217720395

46 Matsuo K, Purushotham S, Jiang B, et al. Survival outcome prediction in cervical cancer: Cox models vs deep-learning model. Am J Obstet Gynecol 2019;220:381.e1-381.e14. doi:10.1016/j.ajog.2018.12.030

47 Merath K, Hyer JM, Mehta R, et al. Use of Machine Learning for Prediction of Patient Risk of Postoperative Complications After Liver, Pancreatic, and Colorectal Surgery. J Gastrointest Surg 2020;24:1843–51. doi:10.1007/s11605-019-04338-2

48 Wong NC, Lam C, Patterson L, et al. Use of machine learning to predict early biochemical recurrence after robot-assisted prostatectomy. BJU Int 2019;123:51–7. doi:10.1111/bju.14477

49 Egger ME, Stevenson M, Bhutiani N, et al. Age and Lymphovascular Invasion Accurately Predict Sentinel Lymph Node Metastasis in T2 Melanoma Patients. Ann Surg Oncol 2019;26:3955–61. doi:10.1245/s10434-019-07690-4

50 Zhou H-F, Lu J, Zhu H-D, et al. Early Warning Models to Estimate the 30-Day Mortality Risk After Stent Placement for Patients with Malignant Biliary Obstruction. Cardiovasc Intervent Radiol 2019;42:1751–9. doi:10.1007/s00270-019-02331-5

51 Alabi RO, Elmusrati M, Sawazaki-Calone I, et al. Machine learning application for prediction of locoregional recurrences in early oral tongue cancer: a Web-based prognostic tool. Virchows Arch 2019;475:489–97. doi:10.1007/s00428-019-02642-5

52 Yan P, Huang R, Hu P, et al. Nomograms for predicting the overall and cause-specific survival in patients with malignant peripheral nerve sheath tumor: a population-based study. J Neurooncol 2019;143:495–503. doi:10.1007/s11060-019-03181-4

53 Fuse K, Uemura S, Tamura S, et al. Patient-based prediction algorithm of relapse after allo-HSCT for acute Leukemia and its usefulness in the decision-making process using a machine learning approach. Cancer Med 2019;8:5058–67. doi:10.1002/cam4.2401

54 Mahmoudian M, Seyednasrollah F, Koivu L, et al. A predictive model of overall survival in patients with metastatic castration-resistant prostate cancer [version 2; peer review: 2 approved]. F1000Res 2019;5:2674. doi:10.12688/f1000research.8192.2

55 Muhlestein WE, Akagi DS, Davies JM, et al. Predicting Inpatient Length of Stay After Brain Tumor Surgery: Developing Machine Learning Ensembles to Improve Predictive Performance. Neurosurgery 2019;85:384–93. doi:10.1093/neuros/nyy343

56 Zhao B, Gabriel RA, Vaida F, et al. Predicting Overall Survival in Patients with Metastatic Rectal Cancer: a Machine Learning Approach. J Gastrointest Surg 2019;24:1165–72. doi:10.1007/s11605-019-04373-z

57 Paik ES, Lee JW, Park JY, et al. Prediction of survival outcomes in patients with epithelial ovarian cancer using machine learning methods. J Gynecol Oncol 2019;30:e65. doi:10.3802/jgo.2019.30.e65

58 Wang X, Zhang Y, Hao S, et al. Prediction of the 1-Year Risk of Incident Lung Cancer: Prospective Study Using Electronic Health Records from the State of Maine. J Med Internet Res 2019;21:e13260. doi:10.2196/13260

59 Li M, Zhan C, Sui X, et al. A Proposal to Reflect Survival Difference and Modify the Staging System for Lung Adenocarcinoma and Squamous Cell Carcinoma: Based on the Machine Learning. Front Oncol 2019;9. doi:10.3389/fonc.2019.00771

60 Nartowt BJ, Hart GR, Roffman DA, et al. Scoring colorectal cancer risk with an artificial neural network based on self-reportable personal health data. PLoS One 2019;14:e0221421. doi:10.1371/journal.pone.0221421

61 Chi S, Li X, Tian Y, et al. Semi-supervised learning to improve generalizability of risk prediction models. J Biomed Inform 2019;92:103117. doi:10.1016/j.jbi.2019.103117

62 Sala Elarre P, Oyaga-Iriarte E, Yu KH, et al. Use of Machine-Learning Algorithms in Intensified Preoperative Therapy of Pancreatic Cancer to Predict Individual Risk of Relapse. Cancers (Basel) 2019;11(5):606. doi:10.3390/cancers11050606
